# Affect and conceptual learning in indoor and green outdoor school environments: Psychophysiological self‐regulation matters

**DOI:** 10.1111/bjep.70052

**Published:** 2025-12-17

**Authors:** Lucia Mason, Libera Y. Mastromatteo, Cecilia Rocchi, Sara Scrimin

**Affiliations:** ^1^ Department of Developmental Psychology and Socialisation University of Padua Padua Italy

**Keywords:** affect, cardiac vagal tone, classroom, conceptual learning, green outdoor, nature, psychophysiological self‐regulation

## Abstract

**Background:**

Research on the role of the physical school environment in areas other than educational psychology has documented the benefits of exposure to nature for cognitive and emotional functioning. Positive effects have been indicated not only after a break in nature in mentally fatigued students but also in students who did not have depleted mental resources when performing cognitive tasks in a green area.

**Aims:**

We investigated the impact of the physical school environment during a single lesson. We also considered psychophysiological self‐regulation as a possible moderator of the relationship between environment and learning.

**Sample:**

We used data from 101 sixth and seventh graders for self‐reported variables. Data about psychophysiological self‐regulation, measured as resting cardiac vagal tone, was available for 83 students.

**Method:**

In a within‐participants research design, we compared the effects of a lesson in the classroom and a lesson in a green urban park close to the school – featuring numerous trees of different sizes, with lush foliage – on participants' affective state, perception of the environmental quality, and conceptual learning.

**Results:**

After the lesson in nature, students reported more positive affect and perceived the park as a higher quality environment compared with the indoor classroom. Students with higher cardiac vagal tone learned more in nature, whereas this individual characteristic did not play a role in the classroom environment.

**Conclusion:**

Passive exposure to nature during a school lesson has a positive affective impact and may also be beneficial for conceptual learning in combination with a higher ability to flexibly adapt to environmental demands.

## INTRODUCTION

Environmental literature has widely documented the positive impact of exposure to nature on cognitive and affective functioning in individuals of different ages (see Mason, Ronconi, et al., 2022; Moll et al., 2022; Nguyen & Walters, 2024 for more recent reviews). Usually, participants' cognitive performance is compared before and after a break in a green space and in an urban area (Schutte et al., 2017), or a break in the green outdoors of a school and in the indoor environment of a classroom (Mygind et al., 2019) to document the beneficial impact of nature, especially on attention performance. In some studies, affective state is also measured using self‐reported positive and negative affect (Berman et al., 2008), or psychophysiological indices (Dettweiler et al., 2022). These studies show that contact with greenness supports a more positive mood and feeling of calm as markers of high arousal or stress decrease. Interestingly, the cognitive and affective benefits were documented not only after a green break in mentally fatigued students (Amicone et al., 2018), but also when students did not have depleted mental resources (Berto et al., 2015). Moreover, research has also documented the impact of long‐term exposure to nature on school achievement by comparing traditional indoor school lessons with lessons in green areas. It is noteworthy that the green environment was not incorporated into the learning activities; that is, exposure to nature was passive (Norwood et al., 2021; Roe & Aspinall, 2011).

We are particularly interested in the effects that nature may have even when considering a single experience with a green area for learning the content of a lesson, not for resting during a break. In the real school context, it is more feasible that students may have an outdoor lesson from time to time. Thus, to investigate the role of the school's physical environment on students' learning and affect has not only theoretical significance for a deeper understanding of a neglected factor in educational psychology research, but also practical implications about why to make them feel calmer and sustain their learning performance (Mason, Manzione, et al., 2022; Mason, Zagni, et al., 2022).

We are also interested in the impact of exposure to nature on early adolescents for three main reasons: (a) they start spending more and more time indoors on the screen of digital devices (smartphones and tablets) to stay connected with social media (Pew Research Center, 2024) and may even have a ‘nature deficit’ (Louv, 2005); (b) their motivation to school learning decreases in the transition from primary to secondary school (Martin & Steinbeck, 2017; Raufelder et al., 2022); and (c) they exhibit wide variability in self‐regulation, reflecting asynchrony between early‐developing subcortical systems that heighten reward sensitivity and still‐maturing prefrontal control circuits. As a result, some youth demonstrate adult‐like inhibitory control, whereas others remain more vulnerable to emotionally charged or rewarding cues due to ongoing brain maturation (Casey et al., 2025).

Learning activities in the outside greenness might promote a deeper engagement and lower levels of perceived and physiological stress (Scott, LoTemplio, et al., 2021; Scott, McDonnel, et al., 2021; Shuda et al., 2020), consequently resulting in greater learning. However, given the individual variability in the ability to self‐regulate and adjust to the environment (Casey et al., 2025), the issue of whether exposure to outside greenness is beneficial for everyone in the same way should be explored.

To advance the scarce psychological research on the impact of passive exposure to nature during a single school lesson, this study aimed to compare affect and learning after a lesson in the greenness and in the classroom in early adolescents, while considering individual differences that could moderate the effects of the greenness on students' performance. Specifically, we took into account their ability to psychophysiologically self‐regulate and adapt to environmental demands, as measured by an objective index of this basic ability for cognitive and emotional functioning, that is, the cardiac vagal tone (CVT). In the next sections, the mechanisms underlying the positive impact of nature are described.

### Cognitive benefit of nature

*Attention Restoration Theory* (ART) accounts for the cognitive benefit of exposure to nature, specifically, attention that is regenerated even after a short contact with the greenness (Kaplan, 1995). As focused attention depends on top‐down control, maintaining it for intense and prolonged time causes mental depletion. Kaplan made a distinction between directed or voluntary attention and fascination or involuntary attention. The first type of attention, which is effortful but limited in capacity, is subject to mental fatigue, so resistance to distractions decreases. The second type of attention is effortless and allows voluntary attention to rest. Natural environments have the potential to reduce the need for voluntary attention and only involve involuntary attention; thus, the inhibitory mechanism of a fatigued person can rest. As a consequence, experience with nature is cognitively regenerating. Indeed, focused attention is a crucial resource in executive functioning and self‐regulation (Kaplan & Berman, 2010) and is at the basis of school performance and success (Diamond, 2013; Rabiner et al., 2016). Evidence of the restorative effects of exposure to nature on attentional systems of children and adolescents is mainly provided in the literature about short‐term green breaks after intense cognitive activity (see Mason, Ronconi, et al., 2022).

Interestingly, for the current study, the cognitive benefits of nature are not only restorative after depletion of mental resources. Even when individuals have not been mentally stressed before exposure to a natural environment, their cognitive resources can be amplified or strengthened above usual performance (Hartig, 2007; Hartig et al., 1996; Moll et al., 2022). In other words, nature also generates the so‐called *instorative* effects, or ‘the capacity to build resources before they get depleted’ (Joye et al., 2024, p. 10). A recent review distinguished between restorative and instorative benefits of exposure to nature (Nguyen & Walters, 2024). For example, Berto et al. (2015) indicated significantly better attention performance in previously non‐fatigued primary school children after 90 min in an alpine wood compared with the classroom (for mindful silence) and the school playground. However, there are also studies that did not find restorative (Stevenson et al., 2019) or instorative effects (Mygind et al., 2019).

### Affective benefits of nature

*Stress Reduction Theory* (SRT, Ulrich, 1981; Ulrich et al., 1991) accounts for the affective benefits of exposure to nature. As a psycho‐evolutionary theory, SRT posits that natural environments have a regenerative effect due to their role over the course of human evolution (Ulrich, 1993; Ulrich & Parsons, 1992). Natural elements rapidly generate a positive affective response as humans are physiologically adapted to natural contexts, in contrast to urban ones. This occurs as natural elements, such as vegetation and water, were important for survival and well‐being during evolution (Ulrich et al., 1991). By generating a positive response, contact with greenness therefore promotes recovery from psychophysiological stress and restoration from stressful experiences. When stress is reduced, feelings of calm and refreshment are experienced, as indicated by emotional states and physiological indicators (Ulrich et al., 1991).

There is ample evidence of the affective benefits of exposure to nature in terms of lower physiological stress response in individuals of different ages, including adolescents, as emerged from systematic reviews and meta‐analyses (Norwood et al., 2019; Twohig‐Bennett & Jones, 2018; Yao et al., 2021). Affect can be measured by using self‐reports (Mason, Manzione, et al., 2022; Roe & Aspinall, 2011) and/or physiological parameters (Berto et al., 2015; Dettweiler et al., 2022; Mason, Manzione, et al., 2022).

### Vagal tone and adjustment to the environment

Learning and acquiring new information to be able to integrate them with pre‐existing knowledge requires the ability to remain focused on a task or lesson for a prolonged period of time. However, sources of distractions within the learning environment can impair the learning process through processing task‐irrelevant information (Escera et al., 2000). This is one of the reasons why learning environments are important (Cayubit, 2022). While green environments have been shown to enhance both cognitive and emotional functioning, students' learning – rather than resting – in such settings may struggle to maintain focus on the lesson for extended periods (Breiner et al., 2018). This challenge is particularly pronounced among students who are not accustomed to spending time in nature. Additionally, adolescence is characterized by an increased attentional focus on socio‐emotional stimuli, particularly peers (Casey et al., 2019). In unfamiliar learning environments, such as parks or gardens, students may feel compelled to monitor their classmates' behaviour potentially perceiving deviations from the norm. This heightened social awareness may contribute to increased distraction, thereby affecting the learning process. Previous studies have shown that new environments (which often include high levels of unpredictability) can hinder the homeostasis of the physiological system which is forced to spend energy to restore balance and adjust. In this condition, fewer resources are available to positively engage in learning activities. Hence, the ability to adapt to new, potentially unpredictable and complex (with colours sounds, and smells) environments might require good physiological self‐regulating ability to help maintain the focus of attention, thus facilitating engagement and learning.

The construct of self‐regulation is fundamental in educational psychology research, as documented in the extensive literature. Despite some differences in describing the phases of the self‐regulated process, the shared definition of the construct refers to the ability to activate and support cognitions, affects, and behaviours oriented towards achieving a goal (Zimmerman, 2015). In this article, we do not address the multiple components of an integrated model of self‐regulation (Blair & Ku, 2022), but focus instead on the basic psychophysiological ability for self‐regulation and adaptation to the environment, which is highly correlated with the ability to inhibit impulses and behaviours (Blair & Raver, 2015). A physiological correlate of this crucial ability is CVT, which is determined by changes in the time intervals between adjacent heartbeats (heart rate variability). As a biological marker of individuals' ability to self‐regulate, CVT reflects the influence of the parasympathetic nervous system on the heart's sinoatrial node (Porges, 2007). The parasympathetic system helps the body maintain (or regain) homeostasis also when exposed to unexpected or stressful stimuli (Li et al., 2009). CVT can buffer the effects of the environment on students' learning, as it is a good index of the ability to adjust to internal and environmental requests through self‐regulation (McCraty & Shaffer, 2015).

Individual differences in resting CVT have been associated with basic self‐regulatory abilities and linked to social, emotional, and cognitive functioning, with higher CVT associated with better outcomes (Graziano & Derefinko, 2013; Miller et al., 2017). Several studies have shown a relationship between higher resting CVT levels and better performance in cognitive tasks requiring executive functions (Thayer et al., 2009), including text comprehension (Zaccoletti et al., 2023) and arithmetical calculation (Mammarella et al., 2023). Indeed, following the neurovisceral integration model (Thayer et al., 2009), a connection exists between the prefrontal cortex and the heart via the central autonomic network and the vagus nerve (Thayer et al., 2009), which justifies the correlation between higher vagal tone and enhanced executive functioning, as well as improved emotional regulation.

In line with this model, research in the school context has documented that students with higher CVT at rest were also able to pay better attention to the lesson when exposed to an unpleasant classroom climate, showing how the physiological index relates to better emotion and self‐regulation (Scrimin et al., 2019; Zagni et al., 2024). Moreover, when comparing primary students' performance in a task of sustained attention indoors and outdoors, it emerged that only in the classroom those with higher vagal tone showed greater focused attention while the physiological measure did not contribute to their performance in the greenness. Despite being mostly exploratory, this work seems to show that inhibitory control is needed more indoors to resist distractions (Mason, Manzione, et al., 2022).

### The present study: research questions and hypotheses

This study is a unique extension of the very few prior investigations on the effects of exposure to nature during a single lesson, which have only been documented in elementary school children, not in preadolescents like in the current study. Moreover, we used CVT at rest as the measure of the individual characteristic of psychophysiological self‐regulation that can moderate the impact of short exposure to nature. We compared the effects of passive contact with greenness during a lesson with those of a lesson in the usual indoor environment of the classroom. Of note is that students were only used to going out to the school courtyard for a short break in the school day. The effects of contact with nature were examined as students' affective state in terms of perceived arousal and valence, perceived environmental quality, and conceptual learning of the lesson content. Students were not in a condition of cognitive resource depletion when they were exposed to nature for the lesson; thus, the study investigated the *instorative* effects of nature (Hartig, 2007; Hartig et al., 1996; Moll et al., 2022) on students' affect, perception of its *instorativeness*, and learning of complex concepts.

Three research questions (RQ) guided the study:

RQ1: Does students' affective state differ in relation to the physical environment where the lesson is taught?
RQ2: Does students' perception of the instorative quality of the two physical environments differ?
RQ3a. Does students' conceptual learning about a science topic differ in relation to the physical environment? RQ3b. Does their psychophysiological self‐regulation moderate this relationship?

Based on research grounded on both theories – SRT and ART – regarding the affective and cognitive benefits of exposure to nature, we hypothesized greater affective state (H1), perception of environmental instorativeness (H2), and conceptual learning (H3a) after the lesson in the green environment. These positive effects were hypothesized since nature induces more emotional well‐being and refreshment (Flom et al., 2011; Haluza et al., 2014; Norwood et al., 2019), is perceived as more instorative than the classroom environment, and strengthens cognitive resources for cognitive processing (Hartig, 2007; Hartig et al., 1996; Nguyen & Walters, 2024).

Moreover, to answer RQ3b, we explored the moderating role of psychophysiological self‐regulation in the relationship between environment and conceptual learning. Students with higher psychophysiological self‐regulation are able to respond flexibly and adaptively to environmental demands. As students living in the city are not used to green environments, they could benefit more from a lesson in the greenness than in the usual indoor classroom, especially if they are better at regulating and adapting to a less‐known learning environment.

## METHOD

### Participants and design

A sensitivity analysis to estimate the adequate number of participants could not be conducted due to the lack of previous studies with the same design. Therefore, a Monte Carlo power analysis based on 500 simulations per sample size was performed using a linear mixed model with fixed effects for environment, vagal tone, and their interaction, and a random effect for participant. Results indicated that ~ 80 participants were required to achieve at least 80% power to detect a medium‐sized interaction effect, with power increasing to nearly 98% at 150 participants. Given the difficulties in recruiting the sample, we aimed at involving around 100 participants.

We initially involved 124 6th and 7th graders attending eight classes in an urban middle school in a mid‐sized town in northeastern Italy. Of the 124 students initially recruited, we excluded the data of five participants due to their absence in one or more sessions; the data of 18 participants were also removed as they had incomplete data for one of the study variables. For 18 students, physiological data were unavailable due to researcher error in failing to start the recording (8 participants) or because the physiological data were not usable (10 participants). Thus, in the analyses, we considered the data of 101 students (*F* = 57; *M*_age_ = 11.45, *SD* = .58) to answer RQ1 and RQ2, and the data of 83 students (*F* = 46; *M*_age_ = 11.44, *SD* = .57) to answer RQ3. Most of the participants, 53.26%, reported belonging to a middle socio‐economic status (SES), while 35.86% to high SES and 10.87% to low SES. As in most studies that do not aim at producing data at a population level, the sample was a convenience one. The participating school had already been involved in previous collaborative projects. The collaboration originated from the shared interest of the school principal, teachers, and parents in applied research aimed at educational development. All students of the classrooms involved participated in the study. The school can be considered representative of urban middle schools in mid‐sized cities of our country for the number of classes and students, and lack of green spaces in its premises. Furthermore, students' socio‐economic status was normally distributed, with most coming from average middle‐class families, reflecting typical characteristics of schools in similar urban contexts (ISTAT, 2024).

Students were involved in a *within‐participants* research design upon their verbal assent and parental written permission. The study was approved by the Institutional Review Board.

### Lessons

All students received the same conditions: for all of them, one lesson took place in the classroom and the other outdoors in a green park close to the school, featuring numerous trees of different sizes, with lush foliage. Relying on teaching materials used in a previous study (Mason et al., 2025), and in accordance with school teachers, the topic of the first lesson for 6th graders was photosynthesis and the impact of deforestation, while the other lesson focused on carbon footprint, carbon cycle, and fossil fuels. The topics of the first and second lessons for 7th graders were air pollution and its impact on our health, and climate change and its impact on agriculture. The lessons were in a counterbalanced order for type of environment and topic. Thus, to exemplify, around half the sixth graders had the first lesson on photosynthesis and the impact of deforestation in the indoor classroom and the lesson on carbon footprint, carbon cycle, and fossil fuels in the outdoor greenness. The other half had the first lesson on carbon footprint, carbon cycle, and fossil fuels in the indoor classroom and the lesson on photosynthesis and deforestation in the outdoor greenness. In this way, one topic was not tied to a particular environment, and the latter was not tied to the first or second lesson.

Importantly, we avoided uncontrolled interfering factors when teaching in four ways: (a) all lessons were taught by a member of the research team (third author) to effectively best ensure uniformity in presenting scientific information across the classes; (b) she presented the content according to a script for each lesson, which detailed each step; (c) the two lessons were independent of each other for conceptual content; and (d) the complexity of the two lessons was the same according to the class teachers who judged them on the basis of the scientific language, novelty of the concepts, and what students already knew about the instructional content. All lessons lasted for 45–50 min.

### Measures

#### Affective state

We used the Self‐Assessment Manikin (SAM; Bradley & Lang, 1994), a non‐verbal pictorial assessment of both arousal (max = 9) and valence (max = 9) of participants' current affective state before and after each lesson in the greenness and the classroom.

#### Pretest

Students' prior knowledge about the topic of each lesson was assessed using 20 multiple‐choice questions with four options (max = 20). As measured by McDonald's *omega*, the reliability of the pre‐tests in the 6th grade was .76 for the topic of photosynthesis and deforestation, and .75 for the topic of carbon footprint, carbon cycle, and fossil fuels. For the 7th grade, the reliability of the pre‐tests was .74 for the topic of air pollution and .78 for the topic of climate change.

#### Post‐test

Participants' conceptual learning from the lesson was assessed using the same 20 questions asked at the pretest a week before. Sample items of questions for the topics in the 6th grade are: ‘What does it mean that plants are autotrophic organisms? (a) Autotrophic organisms depend on other living beings for their food; (b) Autotrophic organisms only eat food that has a vegetal origin; (c) Autotrophic organisms are able to produce their own food; (d) Autotrophic organisms are not able to make photosynthesis’ and ‘What are home activities that contribute most to the carbon footprint? (1) Cleaning floors and dusting the furniture; (2) Cooking inside and outside; (3) Heating and lighting the house; (4) Watching TV or using a computer.’

Sample items of questions for the topics in the 7th grade are: ‘Can nature produce air pollution? 1. No, never; 2. Yes, during a volcanic eruption; 3. Yes, when it rains; 4. Yes, when dead plants decay’ and ‘What damage does climate change cause to agriculture? (1) Climate change increases the stability of weather conditions, making agriculture more predictable; (2) Climate change has no impact on agriculture as plants easily adapt to new conditions; (3) Climate change reduces plant productivity by increasing weather irregularity with drought and other natural disasters; (4) Climate change has no impact on agriculture as climate variations are beneficial for crops.’ As measured by McDonald's *omega*, the reliability of the post‐tests in the 6th grade was .79 for the topic of photosynthesis and deforestation, and .78 for the topic of carbon footprint, carbon cycle, and fossil fuels. For the 7th grade, the reliability of the post‐tests was .77 for the topic of air pollution and .78 for the topic of climate change.

#### Perception of the instorative quality of the physical environment

The Perceived Restorativeness Scale‐Children (PRS‐ch; Berto et al., 2015) was used to assess students' perception of the quality of the environment where the lesson took place. As students were not mentally fatigued when the lessons started and did not need to be restored but rather to gain new knowledge for conceptual learning, we measured their perception of the instorative quality of the environment (Hartig, 2007; Hartig et al., 1996). Such quality refers to benefits in terms of enhancement of available resources. Based on the adult scale (Hartig et al., 1997), the scale for children includes 17 items (0 = completely disagree; 5 = completely agree). Reliability of the scale was .84 for the classroom and .87 for the green environment. Sample items are: ‘This place is interesting’ and ‘This place awakens my curiosity’.

### Psychophysiological self‐regulation (cardiac vagal tone)

In a quiet room of the school, students' cardiac activity was registered at rest for 5 min using a Polar sensor on the thorax. It is a multimodality physiological monitoring device that encodes biological signals in real time (ProComp Infiniti, Thought Technology, Montreal, Canada). The sensor allowed the acquisition of the ECG signal that was then exported in Kubios‐HRV 2.2 software (Kuopio, Finland) to estimate the occurrence of each heartbeat and derive the inter‐beat intervals computed as the difference between successive R‐waves. In addition, the ECG signal was also visually inspected, and a piecewise cubic splines interpolation method was used to correct artefacts. Specifically, this method generates missing or corrupted values into the series of inter‐beat intervals. Successively, the square root of the mean squared differences of successive heartbeats was calculated. Importantly, rMSSD is an index of variation in inter‐beat intervals that is sensitive to short‐term heart period fluctuations; therefore, reflecting parasympathetic activity through the influence of the vagus nerve on the heart (Berntson et al., 1997). In sum, rMSSD can be considered an index of psychophysiological self‐regulation as the ability to adapt and respond to environmental demands.

### Procedure

The study included four sessions. In the first, students' cardiac activity was registered at rest individually. In the second session, they completed a short demographic questionnaire and the pretest. A week later, the third session took place in the green natural area of the park or in the classroom. Before the start of the lesson, participants reported their affective state. After the lesson, they reported their current affect again, performed the post‐test for conceptual learning, and completed the scale about environmental instorativeness. The fourth session took place a week later in the classroom or green natural area, that is, in the other environment than the one of the first lesson.

### Analytical plan

As a first step, we conducted descriptive analyses of the study variables. Next, to examine the impact of the physical environment on affective states (arousal and valence), we ran two linear mixed models (LMM), controlling for the affective state (arousal and valence, respectively) before each lesson. Then, to examine the impact of the physical environment on the perception of its instorative quality, we ran a linear mixed model, controlling for the delta score representing the difference for the valence of the affective state before and after the lesson in each physical environment, where positive scores represent higher valence after the lesson.

Finally, to answer our third research question, that is, the impact of the physical environment on conceptual learning and the moderating role of psychophysiological self‐regulation, we performed a linear mixed model. To account for conceptual learning, we created a delta score (represented by the Greek letter Δ) that reflects the difference between a starting and ending value or parameter. Specifically, the delta represented the overall difference in knowledge levels before (pretest scores) and after (post‐test scores) the lesson in each physical environment.

**FIGURE 1:**
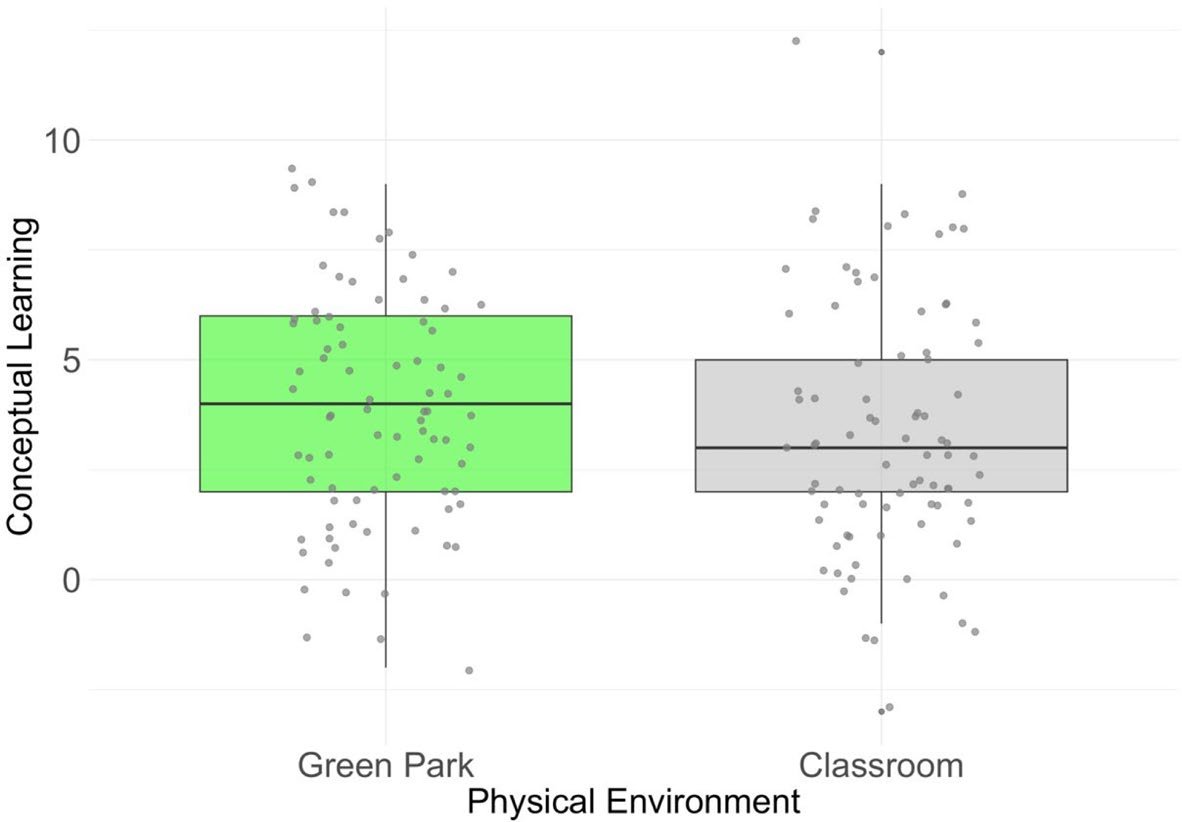
Boxplot of conceptual learning gain (Δ) in the two physical environments.

In all models, we included not only the fixed‐effect factors but also the random effect of participants. Therefore, participants' ID was added as a random factor in all analyses. In all models, the green park condition was used as the reference level. Statistical analyses were performed with the R software (R Development Core Team, 2022), version 4.2.0, using the ‘lmer’ function from the package ‘lme4’ (version 1.1.35.3) for LMMs fitting; the ‘sim_slopes’ and ‘interact_plot’ functions from the package ‘interactions’ (version 1.1.5) for the slope analyses and the resulting interaction figure; the package ‘ggplot2’ (version 3.4.4) for the Figure 1, and the packages ‘sjPlot’ (version 2.8.14) for the tables. Data and codes required to reproduce the analyses are available at the following link: https://osf.io/nr2qb/?view_only=712ec047e04b46b898fc55a21dae8a07.

## RESULTS

Data of all variables were first screened for skewness and kurtosis. Table 1 reports descriptive statistics for all variables.

**TABLE 1:** Descriptive statistics for all variables by physical environment and for the entire sample (*N* = 101).

	Green park			Classroom		
	*M* (*SD*)	Skewness	Kurtosis	*M* (*SD*)	Skewness	Kurtosis
Pretest	9.62 (3.85)	−.11	−.89	12.33 (3.57)	−.78	−.60
Post‐test (conceptual learning)	13.46 (4.17)	−.40	−1.01	15.85 (2.82)	−.46	.30
Affective state–arousal (pre‐lesson)	3.50 (2.28)	.80	−.13	3.73 (2.18)	.62	−.26
Affective state – arousal (post‐lesson)	3.62 (2.34)	.61	−.64	3.56 (2.10)	.42	−.67
Affective state–valence (pre‐lesson)	6.65 (1.85)	−1.02	1.03	6.13 (1.92)	−.24	−.54
Affective state – valence (post‐lesson)	7.16 (1.58)	−.59	−.51	6.12 (1.95)	−.40	−.23
Perceived instorativeness	2.47 (.74)	−.15	−.11	1.38 (.67)	.31	−.34
	***M* (*SD*)**		**Skewness**		**Kurtosis**	
Cardiac vagal tone^a^	53.98 (28.90)		1.02		.71	

^a^ Index of psychophysiological self‐regulation. Only for this variable *N* = 83.

### RQ1: Effects of the environment on affective state

For participants' affective state, both self‐reported arousal and valence were considered. LMM did not reveal the effect of the physical environment (Table 2) on reported arousal (*b* = −.23, *SE* = .19, 95%, CI [−.61, .16], β = −.10, 95%, CI [−.27, .07], *p* = .247).

**TABLE 2:** Linear mixed model for affect –arousal (*N* = 101).

Predictors	Affect – arousal post‐lesson		
	*b* (*SE*)	CI	*p*
(Intercept)	1.07 (.23)	.62, 1.53	**<.001**
Physical environment [classroom]	−.23 (.19)	−.61, .16	.246
Affect – arousal pre‐lesson	.73 (.05)	.63, .83	**<.001**
**Random effects**			
*σ* ^2^	1.88		
*τ*_00 ID_	.39		
ICC	.17		
*N*_ID_	101		
Observations	202		
Marginal *R* ^2^/conditional *R* ^2^	.536/.616		

*Note*: The bold values represent significance.

However, the impact on emotional valence emerged (*b* = −.75, *SE* = .19, 95% CI [−1.13, −.37], β = −.41, 95%, CI [−.61, −.20], *p <* .001). After the lesson in the green natural area, students reported significantly increased positive affect than after the lesson in the classroom (Table 3).

**TABLE 3:** Linear mixed model for affect – valence (*N* = 101).

Predictors	Affect – valence post‐lesson		
	*b* (*SE*)	CI	*p*
(Intercept)	3.47 (.39)	2.71, 4.24	**<.001**
Physical environment [classroom]	−.75 (.19)	−1.13, −.37	**<.001**
Affect – valence pre‐lesson	.55 (.05)	.45, .66	**<.001**
**Random effects**			
*σ* ^2^	1.82		
*τ*_00 ID_	.21		
ICC	.10		
*N*_ID_	101		
Observations	202		
Marginal *R* ^2^/conditional *R* ^2^	.400/.462		

*Note*: The bold values represent significance.

### RQ2: Effects of the environment on the perception of its instorative quality

LMM revealed the main effect of the physical environment (*b* = −1.14, *SE* = .09, 95% CI [−1.31, −.97], β = −1.28, 95%, CI [−1.47, −1.08], *p* < .001). Students clearly perceived the green natural area of the park as more beneficial, that is, instorative than the classroom (Table 4).

**TABLE 4:** Linear mixed model for perceived instorativeness (*N* = 101).

Predictors	Perceived instorativeness		
	*b* (*SE*)	CI	*p*
(Intercept)	2.49 (.07)	2.35 to 2.63	**<.001**
Physical environment [classroom]	−1.14 (.09)	−1.31 to −.97	**<.001**
Affect – Δ valence	−.04 (.03)	−.10 to .02	.**169**
**Random effects**			
*σ* ^2^	.38		
*τ*_00 ID_	.10		
ICC	.22		
*N*_ID_	101		
Observations	202		
Marginal *R* ^2^/conditional *R* ^2^	.398/.528		

*Note*: The bold values represent significance.

### RQ3a and RQ3b: Effects of the environment on conceptual learning and the moderating role of vagal tone

First conceptual learning in the two physical environments was compared to examine whether overall students learned more while in the green natural area as opposed to the classroom. LMM did not reveal a significant main effect of the physical environment (*b* = 1.18, *SE* = .82, 95% CI [−.44, 2.80], β = −.18, 95%, CI [−.46, .10], *p* = .154) (Table 5).

**TABLE 5:** Linear mixed model for conceptual learning gain (*N* = 83).

Predictors	Δ Learning		
	*b* (*SE*)	CI	*p*
(Intercept)	2.71 (.62)	1.48, 3.95	**<.001**
Physical environment [classroom]	1.18 (.82)	−.44, 2.80	.152
Cardiac vagal tone	.02 (.01)	.00, .04	.**038**
Physical environment [classroom] × Cardiac vagal tone	−.03 (.01)	−.06, −.00	.**023**
**Random effects**			
*σ* ^2^	6.17		
*τ*_00 ID_	.98		
ICC	.14		
*N*_ID_	83		
Observations	166		
Marginal *R* ^2^/conditional *R* ^2^	.038/.170		

*Note*: The bold values represent significance.

However, as represented in Figure 1, descriptively, on average students learned more in the green natural area (*M* = 3.87, *SD* = 2.56) than in the classroom (*M* = 3.38, *SD* = 2.83), but standard deviations from the mean scores were higher.

**FIGURE 2:**
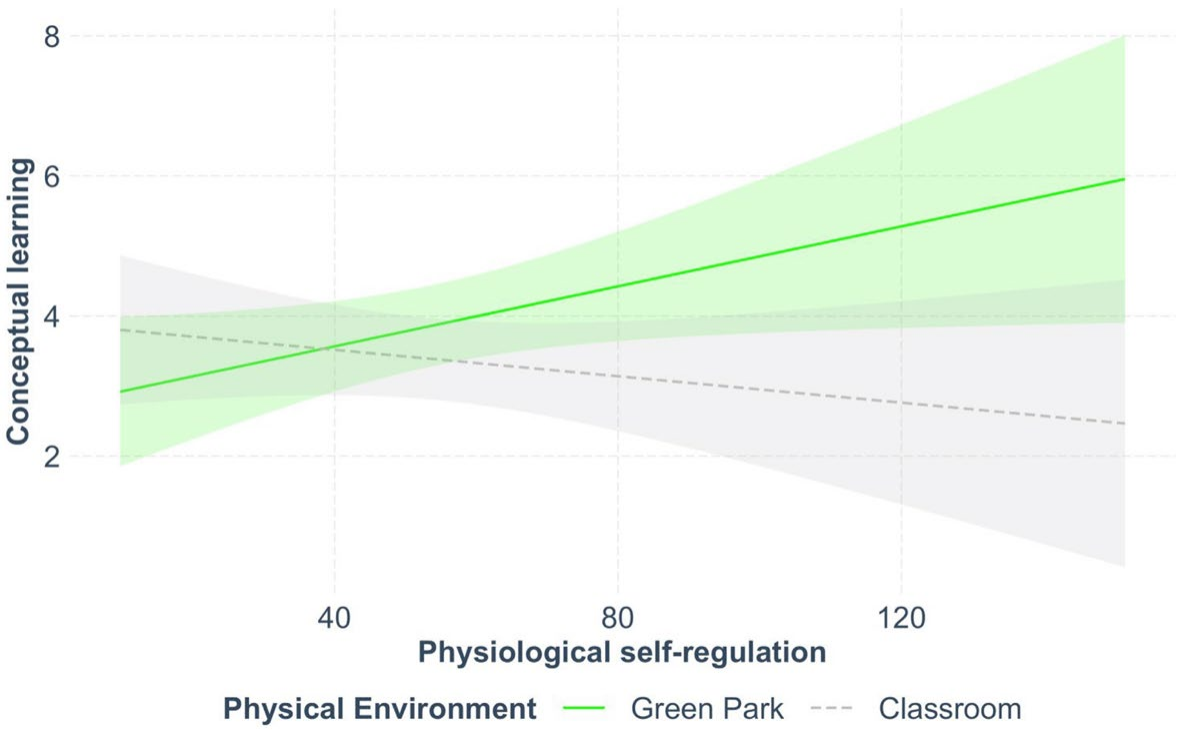
Interactive effect of environment and psychophysiological self‐regulation on conceptual learning gain.

The contribution of psychophysiological self‐regulation was also statistically significant, meaning that, overall, a higher ability to inhibit impulses and behaviours was positively related to greater acquisition of conceptual knowledge (*b* = .02, *SE* = .01, 95% CI [.00, .04], β = .23, 95%, CI [.01, .44], *p* = .038). Most interestingly, the interaction environment × psychophysiological self‐regulation was also statistically significant (*b* = −.03, *SE* = .01, 95% CI [−.06, −.00], β = −.33, 95%, CI [−.61, −.05], *p* = .023). To further probe this interaction effect, we performed a simple slope analysis which revealed that only in the green natural area physiological self‐regulation contributed to improving conceptual learning (*b* = .02, *SE =* .01, *p =* .038), whereas its effect was not significant within the classroom (*b =* −.01, *SE =* .01, *p* = .358). Specifically, as shown in Figure 2, psychophysiological self‐regulation played a significant role in the green natural area of the park as students with greater resting vagal tone, that is, higher ability to self‐regulate and adapt to environmental demands, learned the new scientific concepts better than those with lower vagal tone. However, when learning in the usual environment of the classroom, there was no statistically significant difference between low and high students in psychophysiological self‐regulation.

We also tested interaction effects between environment and affect (both valence and arousal) as well as perceived environmental quality (instorativeness). However, these interactions did not reveal significant moderation effects (all *p*s > .066), suggesting that, in our sample, affect and perceived instorativeness did not substantially alter the relationship between environment and conceptual learning.

## DISCUSSION

This study sought to contribute to the limited research in educational psychology regarding the benefits of contact with natural environments for students' well‐being, cognitive functioning, and school performance. Specifically, to fill the gap in the current literature, based on both Attention Restoration Theory and Stress Reduction Theory, we introduced several novel aspects. (i) We were interested in the learning effect of a short experience with nature as it can more easily take place in the real school context. (ii) We compared students' affective state, perception of environmental quality, and conceptual learning after a lesson, not a break, in the green natural area of a park close to the school, and after a lesson in the usual indoor environment of the classroom. In this way, we investigated the so‐called *instorative* effects of nature, as students were not purposefully fatigued, like in most studies for restorative effects, before being exposed to the green natural area. (iii) We examined students' conceptual learning of the lesson content, not cognitive performance in an unrelated task, and measured learning by using a typical school task, not a lab task.

Our first research question (RQ1) asked whether the physical environment where a regular school lesson takes place would differentiate students' reported affective state. As hypothesized based on SRT, our findings confirm that after the lesson in the green natural area, the valence of participants' affect was significantly more positive than after the classroom lesson. This outcome aligns with previous research on the impact of a short‐term exposure to nature on emotional state relative to an indoor school setting (Mason, Zagni, et al., 2022). Our finding is also in line with investigations on passive long‐term exposure to nature that revealed how green areas can improve mood, among other benefits, in young people (Norwood et al., 2019). We did not find the positive effect of the green environment for arousal. Descriptively, the data indicate a slight overall increase in reported arousal when listening to the lesson in the green natural area. This may suggest that students were not accustomed to spending time in nature and possibly found it challenging to maintain attention while being exposed to a novel environment. Another possible explanation is that reporting personal affective arousal was inherently more difficult compared with valence.

Our second research question (RQ2) asked whether the physical environment in which the regular school lesson takes place would differentiate students' perception of its environmental quality. As hypothesized, participants rated the environmental quality much higher when considering the green natural area of the park, meaning that even when nature is experienced during a regular lesson requiring them to pay attention to the content, they perceive the green natural area as a more instorative environment compared with the classroom, as a recent study also indicated (Mason et al., 2025). This positive environmental perception was previously revealed in school‐age children in both studies on green breaks during a school day after intense cognition (Amicone et al., 2018) and in studies with students who were not fatigued before being at rest in a green natural area (Berto et al., 2015; Mason, Ronconi, et al., 2022).

Our third research question (RQ3a) asked whether the physical environment where a regular school lesson is taught would differentiate students' conceptual learning. Overall, there was no significant difference between students' conceptual learning in the green natural area and in the classroom environment. However, from a descriptive point of view, on average, learning was better in the greenness, but in both environments variability emerged among the students. Such interindividual variability may partly explain the lack of significant difference between the two conditions. Indeed, not everyone benefits from exposure to nature, which could be experienced as a new and challenging environment compared with the well‐known classroom. Previous research on teaching multiple regular lessons along several weeks in nature also revealed no better students' grades compared with lessons in the classroom (Norwood et al., 2021). The literature also shows that some studies did not find a positive impact of the greenness on cognitive performance when using lab attention tasks (Kelz et al., 2015; Müller et al., 2017; Mygind et al., 2019). Moll et al. (2022) also indicated that the instorative effects are smaller than restorative effects after a break and may emerge as robust after repeated exposure to nature over a long time period, not in a short time as considered in the current study.

We can also speculate that affective benefits, or others we did not explore in the current study, are more related to a short exposure to nature during a school lesson. For example, Norwood et al. (2021) reported that during the lessons in nature, students spent more time on task and the teacher had to redirect them less, that this, to stop the lesson less often to reorient their attention or to reprimand them for their inappropriate behaviour compared with the classroom lessons, even if this did not lead to better school achievement.

Our third research question (RQ3b) also asked whether psychophysiological self‐regulation would moderate the relationship between the physical environment and conceptual learning. It emerged that, overall, the higher students' ability of psychophysiological self‐regulation, the greater their acquisition of new scientific knowledge. This is a finding aligned with research on the relationship between resting vagal tone and academic performance, for instance, comprehension of written (Scrimin et al., 2018; Zaccoletti et al., 2023) and oral (Scrimin et al., 2017) texts. However, the findings about conceptual learning should be interpreted in light of the interaction between environment and vagal tone as this psychophysiological individual difference moderated the effect of the environment on conceptual learning, partially confirming what we could expect based on ART. Interestingly, in the green natural area of the park, students with higher ability to respond and adapt to environmental demands performed better than those with lower ability. In other words, only students with greater psychophysiological self‐regulation learned more in the green environment, meaning that these students were better able to maintain focused attention on the science content despite being in a novel, stimulating, and potentially unpredictable outdoor context.

This result can be interpreted in light of theoretical models linking cardiac vagal control to adaptive self‐regulation. According to Thayer and colleagues' neurovisceral integration model (Thayer et al., 2009, 2012), higher resting HRV reflects greater functional capacity of prefrontal inhibitory networks that support flexible adjustment to environmental demands and maintenance of goal‐directed behaviour. Thus, individuals with higher HRV at rest are more capable of sustaining attention and emotional stability when facing novel or uncertain contexts. Our finding aligns with this framework, indicating that only students with higher resting HRV – that is, those with greater psychophysiological flexibility – were able to optimally benefit from the learning opportunities provided by the green natural setting. This interpretation complements the literature emphasizing that psychophysiological self‐regulation helps maintain homeostasis and a state of receptive calm in potentially unpredictable environments (Corcoran et al., 2021), but refines it by highlighting that such regulatory capacity may particularly benefit individuals with higher baseline flexibility.

For urban children like in our study, a green natural area as a school environment for the first time might also have been distracting due to its natural sounds (birds) and small animals that were around. Moreover, adolescents might have been more focused on their peers' behaviour, which was much more unpredictable than usual given the lack of familiarity with the green natural area as the setting for a school lesson. Thus, it is theoretically legitimate that students with higher vagal tone indexing their basic ability to stay concentrated if it is required by the environmental demand show better learning in the green natural area. In contrast, in the usual and very familiar environment of the classroom, in general students are already used to paying attention and listening to the teacher. This may explain why their ability to self‐regulate at the psychophysiological level was much less relevant indoors than outdoors.

The current finding apparently seems to be at odds with the Mason, Zagni, et al. (2022) outcomes of a study in which higher psychophysiological self‐regulation was a resource for better attention performance only in the classroom environment, not in the school green garden. However, it should be noted that in the Mason, Zagni, et al. (2022) study, students were younger (elementary school) and less captured by socio‐emotional stimuli coming from peers. Moreover, elementary school students were used to spending time in the school garden, including long classroom hours. As such, the green natural area represented a much more predictable and less challenging environment for elementary than middle school students. Lastly, the cognitive task in the Mason, Zagni, et al. (2022) study was a typical lab attention task, that is, unrelated to the school activity performed prior to it. This represents a notable difference from the present study, which required, in order to learn well, paying attention to the entire lesson and not just an attention task that lasts a few minutes.

From a more general perspective, greater resting vagal tone –as derived by heart rate variability– has been documented as a predictor of students' conceptual learning (Scrimin et al., 2018), metacognitive judgements of learning (Meessen et al., 2018), engagement in learning activities when classroom climate is negative (Mastromatteo et al., 2023), cooperative behaviour (Mastromatteo et al., 2024; Zagni et al., 2024), and preschoolers' listening comprehension (Scrimin et al., 2017). In the present study, this index of physiological regulation explains part of the reason why not all students benefit from lessons taking place in the green environment. That is, when this environment is new and perceived as challenging, a good regulatory ability is needed to pay attention to a school lesson and indirectly benefit from exposure to nature. For this reason, interventions fostering students' physiological self‐regulations should be promoted in schools.

Specifically, psychophysiological self‐regulation can be fosteed in children through structured, age‐appropriate methods such as controlled breathing exercises, guided relaxation, and simple mindfulness practices. For example, brief sessions of diaphragmatic breathing have been shown to enhance parasympathetic activity and heart rate variability, supporting stress regulation and emotional balance (Thayer et al., 2012). Similarly, mindfulness‐based interventions adapted for schools have demonstrated benefits for attention regulation, emotion management, and overall well‐being (Zenner et al., 2014). Progressive muscle relaxation and biofeedback training are further approaches that have been effectively used to help children recognize bodily stress signals and develop strategies for calming physiological arousal (McGuigan & Lehrer, 2021). Integrating such self‐regulation techniques into classroom routines can support students in recovering from stress, sustaining focus, and improving their learning environment's overall ecological quality (Blair & Raver, 2015). From the present work, we add to this list showing how such practices might also help students benefit more from learning in a green natural area.

### Limitations

Like any study, the current one has some limitations that should be taken into account. First, the sample size was limited and did not allow for the inclusion of all the outcome variables in a single analytical model. Future research with a larger number of participants and a suitable multivariate framework could further examine the relationships among the variables.

Second, the lessons were not taught by the class teachers but by a member of the research team to ensure uniformity of the information provided and the teaching style across the various classes. Thus, the ecological validity of the study is suboptimal. A step forward in this line of research is to involve teachers in giving regular lessons on the basis of shared scripts. In this way, more generalizable findings will be available.

Third, we only measured vagal tone at rest and not during the lessons in the two environments. As physiological measures are collected individually, a simultaneous recording of cardiac activity requires multiple portable devices available at the same time, which is not always feasible. This constraint also explains why limited samples usually characterize studies based on students' physiological measures (Berto et al., 2015; Dettweiler et al., 2022; Mygind et al., 2019). Future research aimed at examining objective measures of stress reduction should contribute to the current literature by focusing not only on physiological parameters at rest but also during the learning activity and adopting a multimethod approach to reveal affective and cognitive benefits according to the integration of SRT and ART (Scott, McDonnel, et al., 2021), grounded on the neurovisceral model by Thayer et al. (2009). Fourth, and related to the previous issue, we did not include a self‐reported measure of self‐regulation (e.g., Helle et al., 2013), which would have helped to validate and potentially augment the psychophysiological measure as the latter is, to some extent, ‘hypothetical’ and inferred. In the next studies, self‐reported measures should complement and integrate psychophysiological measures.

Fifth, we did not consider participants' motivational factors in relation to academic learning. Students who are more demotivated and disengaged towards school activities, for example, might benefit more from exposure to a green environment during teaching, as nature makes them feel calm and serene, promoting emotional well‐being, which is a resource for learning processes and outcomes (Norwood et al., 2021; Vella‐Brodrick & Gilowska, 2022).

## CONCLUSIONS

Despite these limitations, the study contributes to current research on the benefits of nature by showing that even the first, short, and passive exposure to a green natural area during a regular school lesson can improve students' affective state, lead them to perceive the natural environment as more restorative, and be beneficial for their conceptual learning in combination with a higher ability to adapt and respond to the environmental demands. Overall, the findings add to the current research that mainly reports benefits of green breaks in a school day (Mason, Ronconi, et al., 2022) or long‐term exposure to nature (Norwood et al., 2021; Vella‐Brodrick & Gilowska, 2022).

Specifically, from a theoretical point of view, the study provides evidence of the advantages of being in nature to feel a more positive mood and better learn the content of a lesson on complex scientific concepts, thus adding to the literature that shows *instorative* effects of passive exposure to a green area (Moll et al., 2022; Nguyen & Walters, 2024). We therefore contribute to research on *instoration* as building stronger resources before they are depleted (Hartig, 2007; Hartig et al., 1996; Joye et al., 2024), supporting a wider view of the cognitive and affective benefits of nature as expected by the aforementioned ART and SRT.

In particular, the study suggests that the individual difference of resting psychophysiological self‐regulation is a resource when learning in a new unpredictable setting like the green natural area of a park that is used to teach a regular lesson. In fact, a natural space that becomes a learning environment for the first time requires students' good ability to respond appropriately at the basic level, being excited at the beginning but then calm and concentrated with focused attention to learn new complex concepts. In this regard, it should be pointed out that educational interventions to enhance social and emotional learning contribute to increasing psychophysiological self‐regulation from pre‐K to grade 12 (Blewitt et al., 2024) as aforementioned.

From the practical point of view, the study suggests a feasible way to respond to today's children's ‘indoor‐ification’ (Sobel, 2014) as they spend less and less time outdoors and most of their free time indoors interacting with technological devices. Thus, they have less and less contact with nature, if not a nature deficit (Louv, 2005). It is therefore educationally important to give students opportunities to be exposed to green natural areas during school activities. Such opportunities, which might be at relatively low cost in some cases, have the potential to contribute to students' greater affect and learning in combination with individual differences, like psychophysiological self‐regulation.

## AUTHOR CONTRIBUTIONS

**Lucia Mason:** Conceptualization; methodology; funding acquisition; writing – original draft; supervision; project administration; writing – review and editing. **Libera Y. Mastromatteo:** Data curation; validation; visualization; formal analysis; software. **Cecilia Rocchi:** Investigation; resources; data curation; methodology. **Sara Scrimin:** Writing – original draft; methodology; supervision; data curation; conceptualization; writing – review and editing.

## CONFLICT OF INTEREST STATEMENT

The authors declare they they have no conflict of interest.

## Data Availability

Data and codes are available at the Open Science Framework (OSF): https://osf.io/nr2qb/?view_only=712ec047e04b46b898fc55a21dae8a07.
